# Modeling students’ performance using graph convolutional networks

**DOI:** 10.1007/s40747-022-00647-3

**Published:** 2022-01-18

**Authors:** Ahmed A. Mubarak, Han Cao, Ibrahim M. Hezam, Fei Hao

**Affiliations:** 1 School of Computer and Science, Shaanxi Normal University, Xian, 710119 China; 2 Department of Information Technology, Ibb University, Ibb, Yemen; 3 Statistics and Operations Research Department, College of Sciences, King Saud University, Riyadh, Saudi Arabia; 4 Department of Computer Science, University of Exeter, Exeter, Ex4 4QF UK

**Keywords:** Course MOOC, Graph convolutional network, Heterogeneous knowledge graph, Semi-classification, Prediction

## Abstract

Many models were recently proposed to classify students, relying on a large amount of pre-labeled data to verify their classification effectiveness. However, those models lack to accurately classify students into various behavioral patterns, employing nominal class labels, rather than ordinal ones. Meanwhile, such models cannot analyze high-dimensional learning behaviors among learners according to students’ interaction with course videos. Since online learning data are huge, the main challenges associated with data are insufficient labeling and classification using nominal class labels. In this study, we proposed a model based on Graph Convolutional Network, as a semi-supervised classification task to classify students’ engagement in various behavioral patterns. First, we proposed a label function to label datasets instead of manual labeling, in which input and output data are labeled for classification to provide a learning foundation for future data processing. Accordingly, we hypothesized four behavioral patterns, namely (“High-engagement”, “Normal-engagement”, “At-risk”, and “Potential-At-risk”) based on students' engagement with course videos and their performance on the assessments/quizzes conducted after. Then, we built a heterogeneous knowledge graph representing learners, course videos as entities, and capturing semantic relationships among students according to shared knowledge concepts in videos. Our model intrinsically works for heterogeneous knowledge graphs as a semi-supervised node classification task. It was evaluated on a real-world dataset across multiple settings to achieve a better predictive classification model. Experiment results showed that the proposed model can predict with an accuracy of 84% and an f1-score of 78% compared to baseline approaches.

## Introduction

Recently, online learning platforms have become a modern environment for educational process advancement, most universities have headed to leverage these platforms in order for the educational process to continue, especially in the hard times of the COVID-19 outbreak. The online learning platforms provide courses in form of video lectures, discussion forums, assessment online, and even live video discussions. Video lectures play a prominent role in online courses and cover all course concepts. Learners spend most of their time interacting with video lectures. However, learners may ignore or skip some videos of the course looking for some specific concepts or knowledge to achieve their goals based on their personal needs. Accordingly, each student has his own learning style, which affects his way of getting, understanding, and perceiving information in learning environments. The differences in learning behaviors and learning styles of students have led to the rise of a wide variety of researchable problems about students’ behavior in different educational contexts [1].

Fortunately, online learning environments provide a huge volume of students’ data educational various that have established novel research direction called Learning Analytics (LA), which have primarily focused on resolving significant educational problems, such as tracking student’s performance, reducing high dropout rates among enrolled students, and improving their learning environments [2]. In this respect, many efforts were made including behavior prediction [3], course recommendations [4], understanding user intentions [5], early prediction of students who dropout [6], assessment of students’ performance [7], and tracing knowledge [8]. Some related studies indicated that course completion rate is lower than 5% [9] and the rate ranges between 0.7 and 52.1% with a median value of 12.6% [10]. Other studies indicated that completion of online courses can be predicted by analysis of students’ behaviors during video-watching [11, 12]. They analyzed the viewing behavior to obtain useful feedback. These feedbacks serve to enhance the effectiveness of video lectures, to predict student's performance, and likewise to improve the learning process. They also indicated that the study of the learning behaviors result from students’ interaction with course videos related to metacognition field and self-organized learning. In line with this, many of these studies have emphasized the significance of learner video-viewing behavior as a key feature for dropout status predicting. For instance, Lan et al. [13] employed behavioral data to model learners’ engagement during viewing lecture videos and connected it to their learning outcomes. The authors selected behaviors interactive such as number of pauses, number of rewinds, number of fast-forwarding, and average playback rate. They indicated that it was possible to measure student engagement only with their log data. However, those studies ignored the clicks behavior vis a vis learning styles and individual differences, which can be an important feature to improve video-viewing behavior analysis. Kim et al. [14] examined learners' learning patterns engaging in open courses through the lens of self-regulated learning. The authors connected learner engagement with their ability to self-regulate through time and resource management skills. The results demonstrated learners, who were more engaged in social interaction, with high levels of self-regulated learning skills. Rybakova and Witte [15] observed that low-engaged students only view the course content. Kim et al. [16] referred to proxy variables identifying students’ self-regulated learning by employing students’ log data in an asynchronous learning course video. Results indicated that their model can identify at-risk students with low engagement in the early days of the online course.

In a MOOCs’ environment, individual skills among students are clearly evident in terms of learning duration, elected learning content, and their learning style. Among all these skills, learning style is a significant factor that impacts students’ individual differences [17]. Also, video-viewing behavior analytics can convert as an important advantage in the learning process to classify students into different behavioral patterns based on their engagement level.

In line with that, researchers have made relevant contributions concerning how to classify students into different levels according to their own learning styles and engagement level with educational content. They employed machine/deep learning techniques to students' performance modeling, and they showed that there is a positive relationship between students’ engagement and academic performance with higher engagement levels associated with better grades [18–20]. Meanwhile, many previous works studied various methods of defining students' engagement levels using different engagement metrics. Some researchers proposed a three-level model [21, 22] which classified students' levels as either high, nominal, or low. Others assumed a five-level model for classifying students into one of the following categories: authentic engagement, ritual compliance, passive compliance, retreatism, and rebellion [23]. Kamath [21] classified students into three levels based on their engagement using image recognition as the basis of their classification by constructing a custom dataset of images representing various engagement levels. However, this is as only useful in a real-time scenario, and their models evaluated students’ engagement based on in-classroom interaction, making it more difficult to adopt in an online environment. These studies provided approaches for intelligent classification and prediction of students’ outcomes. However, such approaches were not based on large-scale online learning platforms. Additionally, these classification approaches analyze only the surface student characteristics (e.g., stages of engagement embodied within the motivational perspective, or the large-grained behavioral and the emotional perspectives), but they are unable to accurately classify learners into different behavioral patterns based on video-viewing behavior. Furthermore, the algorithms used require enormous amounts of labeling data for classification, which is unrealistic for online learning data. Because, these data are lacking labeled and classification specifically nominal class labels.

For this purpose, a significant aspect of this study aims to come up with novel methods to investigate students’ interactive engagement that can be detected by investigating student–video interaction and their performance on the assessments/quizzes conducted after. Thus, students are classified according to the identified learning behavioral patterns, and then, those who are mostly at-risk of dropping out of the course are identified. Therefore, instructors can then make interventions based on student classification by providing more intensive interventions for the students who are at a higher risk, or providing lighter interventions for the students who are at a lower risk. In line with the above discussion, the first step is to build a heterogeneous knowledge graph to model the relation among different entities (students, videos) and identify the link among students on the basis of the concept of knowledge related to video. Based on the heterogeneous knowledge graph constructed, supervised classification learning algorithms cannot be employed due to the huge amount of graph data and high labeling cost. Therefore, we seek to employ semi‐supervised learning classify students’ engagement levels in online courses.

Semi-supervised classification methods can be expressed as the incorporation of unsupervised and supervised approaches [24], and can employ both of them. Many utilized semi-supervised learning techniques have several types such as self-training, co-training, transductive support vector machines, and graph-based techniques, which incorporate labeled and unlabeled data to increase performance accuracy of prediction [24, 25]. Semi‐supervised learning utilizes a set of labeled and unlabeled data and seeks to converge and predict data points. Utilizing both classes of data is due to the enormous amounts of unlabeled data, while labeled data are difficult to find, and it is a very expensive task to label the unlabeled data [26]. In graph-based techniques, nodes and edges are used to model data structure as a graph structure. Nodes define labeled and unlabeled datasets and edges represent similarities between nodes [27].

According to above facts, node classification in graphs is an unsupervised learning task that indicates clustering of nodes with similar characteristics. Uncovering the labels for a small percentage of nodes transforms the unsupervised node classification task into a semi-supervised learning task. Graph-based semi-supervised learning methods aim to predict the labels of those unlabeled nodes by utilizing label dependency information reflected by known label information.

In accordance with that, we propose a novel classification approach based on Graph Convolution Networks (GCNs) as semi-supervised learning tasks for classification on large-scale online learning data.

The proposed model intrinsically works for heterogeneous knowledge graphs to classify students’ learning styles and predict their performance in online courses. The proposed model also works to solve the gradient vanishing problem in deep GCNs by adding direct mapping between different layers of the deep GCNs to ensure that the *L* + 1 layer network contains more image information than the *L* layer. Therefore, GCNs predict the classes through the ‘message-passing’ mechanism, i.e., they aggregate the semantic representations between each node and its neighbors at each layer to generate the final-layer predictions. Then, prediction of students’ performance is formalized in a semi-supervised scenario to classify them at different levels.

In this method, four behavioral patterns, i.e., High-engagement, Normal-engagement, Potential-At-risk, and At-risk, were adopted as the classification criteria (more details are presented in Sect. “Classification modes”). First, we analyzed students' interaction data in the course using a proposed algorithm called the feature extraction process to extract features matrix and then apply proposed algorithm called the labeling function to label data according to the identified classifications (Sect. "Dataset description"). Then, labeled training data were fed to the GCNs’ model, which were used as training samples to train the model to learn the high-dimensional student interaction features based on a heterogeneous knowledge graph and classify students accurately. The trained model was applied on test data (unseen data) to classify students’ engagement. The results show that the method proposed in this paper is superior to the traditional methods.

The main contributions in this paper are:

- Constructing a heterogeneous knowledge graph to represent various complex interactions among different types of entities (student, videos) in an MOOC course.
- We identified four engagement levels, which are High-engagement, Normal-engagement, Potential-At-risk, and At-risk.
- By data programming method, we propose label function for labeling dataset in different engagement levels as ground truth.
- Formulating prediction of students' performance as a semi-supervised node classification on heterogeneous knowledge graph that snapshots the underlying relationship between course videos and students.
- Proposing a graph convolutional network model to classify students' performance in the course according to their interaction with course videos, which intrinsically work for heterogeneous networks.

The rest of this work is organized as follows: Sect. "Classification modes" introduces a classification modes and problem statement. The proposed model is presented in Sect. "Classification proposed model". Section "Discussion Results and Evaluation of the Model" discusses the experiment and the results. Finally, the conclusion and suggestions for future work are presented in Sect. "Conclusion and future works".

## Classification modes

Students’ engagement is a multi-faceted concept and can be measured differently depending on learning contexts and objectives [29]. Angrave et al. [30] referred to the identification of reliable measures representing various aspects of students’ participation in learning environments. In online learning, courses are traditionally organized about video lectures. Consequently, students’ engagement can be measured throughout their video-watching. The study of video-watching behaviors in online learning platforms correlates to the field of self-regulated learning. In this regard, the researchers on online learning stated that learners have various learning patterns, and thus, individual levels should be investigated by video-watching behaviors analyzed, assuming the heterogeneity of learners [31]. Therefore, learners’ traces have been employed during video-viewing to measure learner engagement by studying explicit events on video (i.e., play, pause, seek forward/backward, and so on). Although far from perfect, explicit events are a good proxy for reflecting different patterns of engagement that analyzers can employ to discover whether a student is really engaged. To provide insights to online learning instructors about how learners learn differently from online learning course videos that can meet the individual needs of learners with diverse learning patterns. In that regard, we need to identify various classification modes to classify learners according to their own learning styles. In this study, we hypothesize four behavioral patterns that can identify students’ interaction behavioral indications and can differentiate them as High-engagement, Normal-engagement, Potential-At-risk, and At-risk. The mode at which students engage in the course can be seen by considering features involving interaction-related features (events while watching-video such as play, pause, seek forward/backward, speed change, move slide, change volume, and so on) and effort-related features (performing week quizzes and a number of attempts for each quiz) (see Table 1). Therefore, students can be classified into any of the proposed modes based on these features inferred from their educational data collected in online learning platforms.

**Table 1:** Description classification modes

Mode	Engagement activities
High-engagement	Students who watched all videos and at the same time took weekly quizzes with one attempt
Normal-engagement	Students who watched all videos but did not watch most of the whole videos and have worked on weekly quizzes with at least three attempts
Potential-At-risk	Students who watched most of the videos, but at a time that does not exceed half the video's actual time and their performance for weekly quizzes was not done properly, and some students rarely did it with attempts reaching the highest limit (five times) to re-quiz
At-risk	Students who watched some videos for a short time and did not take weekly quizzes

The first column in Table 1 shows the four modes proposed of engagement behaviors, while the second column shows characteristic descriptors for each mode of engagement behaviors. The first component mostly includes students who interacted with the videos and fired events while watching to understand each part of the videos. Meanwhile, it is possible that these students were more effective in doing other activities such as reading, tracking-related topics, and forum discussion. Additionally, they performed the weekly tasks and duties and passed the test on the first attempt. This assures that they work at a high pace throughout the course. This class is called “High-engagement”. Similarly, the second component involves students who watched all videos, but did not watch most of the whole videos. It is possible that these students were less effective in doing other activities, and the main factor for gaining information was watching the video without delving into the relevant topic, which indicates that their performance in the weekly tests was done in more than one attempt (at least three); this class falls under “Normal-engagement”.

In the Potential-At-Risk mode of engagement, students receive information while video-viewing, without doing anything else related to learning. We conceptualize that these students may aim to search for information by watching certain parts of the video, but at a time that does not exceed half the video's actual time. Therefore, they do not like to continue watching the educational videos. In addition, they may focus their attention on a particular part to pass the exam and do the weekly tasks, but they fail in the first attempts as a result of not having acquired the required information.

From an At-risk of dropout mode perspective, students may view some videos from the first week that highlight the goals of the course, and then decide whether to continue learning or not. In fact, during the first half of the course, vulnerable students often tend to decrease their interactions with the course activities, or they even might leave it completely. Therefore, these changes in behavior or interaction are considered as alerts that show early signs of failure.

To formulate these hypotheses practically, we proposed a data programming-based algorithm, called the labeling function, which can label the data to the identified classifications that can be adopted as ground truth to be compared to the class label that the model predicted (more details are presented in Sect. "Dataset description"). The algorithm was applied to the data to build ground truth before it was considered as input data of the proposed model and to provide a learning foundation for future data processing.

## Classification proposed model

**Fig. 1:**
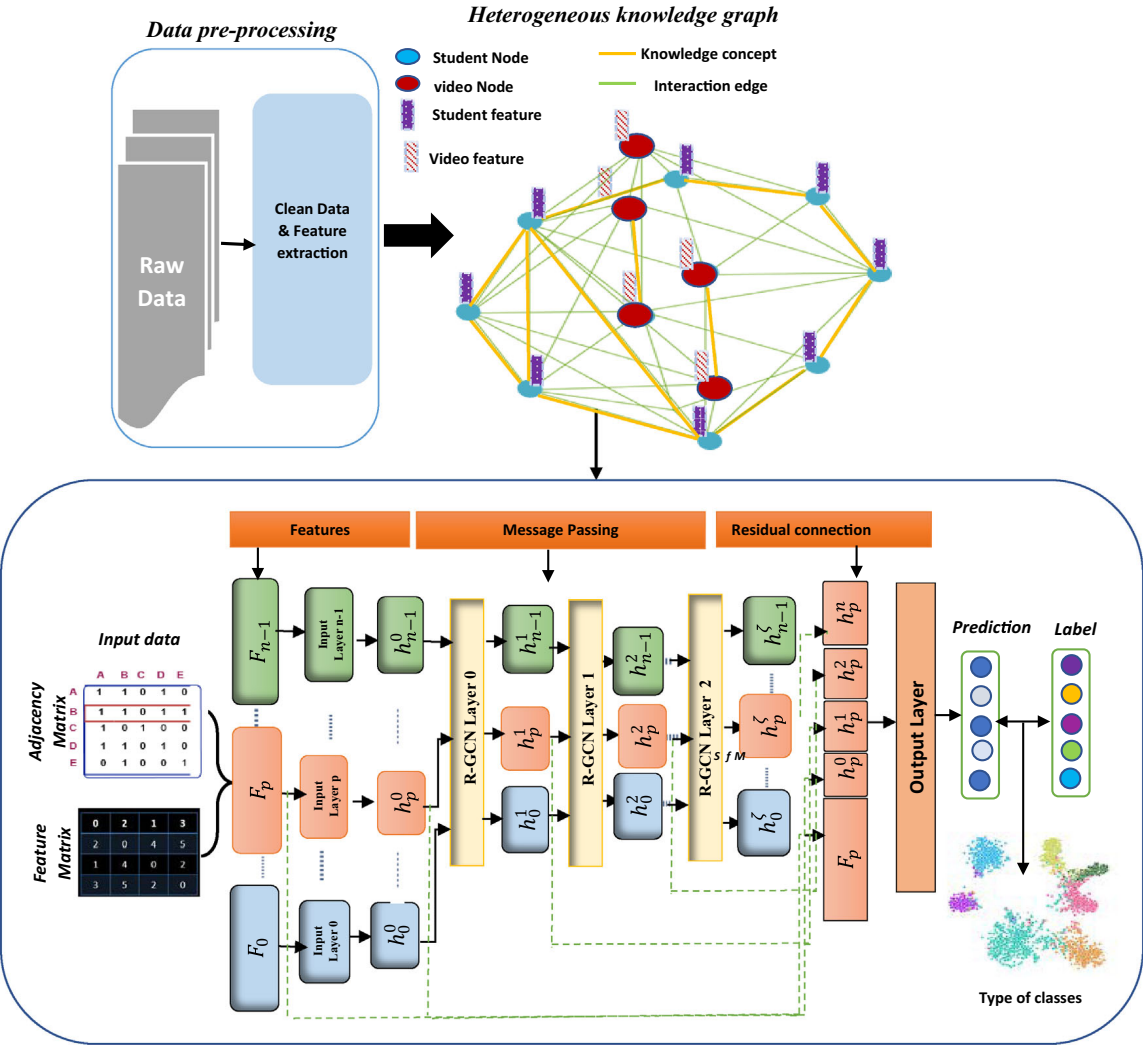
Diagram of proposed Model structure

In this section, the proposed framework of our approach is explained. It consists of three main modules, which are feature extraction, heterogeneous knowledge graph representation, and application of GCN on semi-supervised learning tasks, as shown in Fig. 1. We explained all the notations used in this work in Table 2.

**Table 2:** Notations and explanations

Notation	Explanation
Symbols of the constructed structured heterogeneous knowledge graph	
$S$	The set of nodes of students’ indexing by *s* = {1, 2, 3, …., *K*}
$$ {X}_{s}$$	Matrix of students' interaction data with the course
$$ V$$	The set of videos nodes indexing by *v* = {1, 2, 3, …, *m*}
${ X}_{v}$	Matrix of videos features
${ d}_{v}$	The dimension size of video feature vector
${ d}_{s}$	The dimension size of students' feature vector
$$ G$$	A heterogeneous knowledge graph
$E$	Set of edges in *G* network
Symbols of graph convolutional network model	
${ A}_{i,j}$	Adjacency matrix where $i$ is node number, $j$ is Node *i*’s neighbor
$$ N$$	Number of nodes in *G* network
${\rm X}$	Features’ matrix in *G* network
$$ {N}_{u}$$	Set of unlabeled nodes
$d$	The dimension size of each node's feature
$$ Y$$	Number of classes in output layer
$\stackrel{\sim }{ D}$	Degree matrix based on *G* network structure
${ I}_{n}$	The identity matrix
${ H}^{\left(l\right)}$	Feature matrix given by *l*th layer in GCN
${W}^{\left(l\right)}$	Weights of *l*th GCN layer
$\sigma$	$ReLU\left(a\right)=\mathrm{max}(0,a)$ function
${ g}_{\theta }=diag(\theta )$	A filter on spectral domain
$\theta$	Parameter $\in {R}^{N}$ in the Fourier domain
$$ U$$	The eigenvectors matrix
${ f}_{\mathrm{softmax}}$	SoftMax function
$$ {Z}_{k}^{n}$$	The GCN output layer that indicates label prediction

We seek to build a model to classify students’ level of engagement in a course in the current week, given their interaction data with course videos in previous weeks. One stage is to construct students’ interactions with course videos as a heterogeneous graph with representing features related to each student and video (clickstream data). Then, the adjacency matrix ${A}_{i,j}\in {\mathbb{R}}^{k\times k}$ from a graph is extracted beside the features matrix for students as input fed to classification model. In this respect, we consider the problem of classifying students as a semi-supervised node classification problem on a graph where labels are only available for a small subset in graphs to unknown class labels.

Formally, we can define this problem as follows. Given students, videos in graph $G=(X,E,S,V)$ and ${s}_{xi}$ is the attribute vector associated with the vertex${S}_{i}\in X$, where $X$ is a finite set of vertices in the given graph G. Let $Y =\{{y}_{1}; {y}_{2}; {y}_{3};\dots .;{y}_{l} \}$ be the set of $l$ labels. The training dataset is defined by${D}_{t}=\{\left({G}_{1}; {y}_{1}\right); \left({G}_{2}; {y}_{2}\right);\dots .; \left({G}_{t}; {y}_{t}\right)\}$, where $t$ is the total number of training samples.

More formally, considering the target student $S$ with his corresponding interactive data in the course and his correlation with peers $Pr$ in graph, the goal is to calculate the student's engaged level. Therefore, the classifying function $f$ is learned and used to generate a classification list of engagement levels $C$, such that

$$ f{ }:{ }\left( {S,{ }Pr} \right){ } \to { }\{ yi{ }|yi{ } \in { }C,{ }i{ } < { }C\} . $$

### Feature extraction

Based on the historical data of the MOOC course, we extract features of entities and analyze the different relationships (e.g., student: ${S}_{1}$ and ${S}_{2}$ watched video: ${V}_{1}$ and video:${ V}_{2}$; or student S1 watched two videos. This first behavior implies a relation between two students, and the other behavior denotes a relation between two videos in the same knowledge concept). Therefore, to model MOOC course data structure as graph for each an online course, we have a set of $m$ videos nodes denoted as $V=\{{v}_{1},{v}_{2},\dots .,{v}_{m}\}$. For each video${V}_{v}$, we presume some related features such as name of knowledge concept and events (e.g., play, pause, stop, forward, and backward) that can be transformed as the vector ${X}_{v} \epsilon {\mathbb{R}}^{{d}_{v}}$ with ${d}_{v}$ being the dimension size after encoding the video features. In a likely manner, there are $K$ students enrolled in this course, which we point out as nodes $S=\{{s}_{1},{s}_{2},\dots .,{s}_{K}\}$. Each student ${S}_{s}$ has his/her interaction data with video transformed into the vector ${X}_{s} \epsilon {\mathbb{R}}^{{d}_{s}}$ with ${d}_{s}$ is the dimension size of the vector resulted from encoding students' interaction data with video. Moreover, the behavioral data for each student ${s}_{i}$ who watched course videos are represented as ${X}_{i}=[{X}_{i}^{1},{X}_{i}^{2},\dots .{X}_{i}^{m}]$ where ${X}_{i}^{m}\epsilon {\mathbb{R}}^{{d}_{s}}$ represents encoding of the behavior for student ${s}_{i}$ during the video ${v}_{m}$, *m* represents the number of videos for which behavioral data were collected, and ${\mathbb{R}}^{{d}_{s}}$ is the dimension of the encoded student's behavior per video. The pseudo-code of the feature extraction process is shown in Algorithm 1 (Table 3).
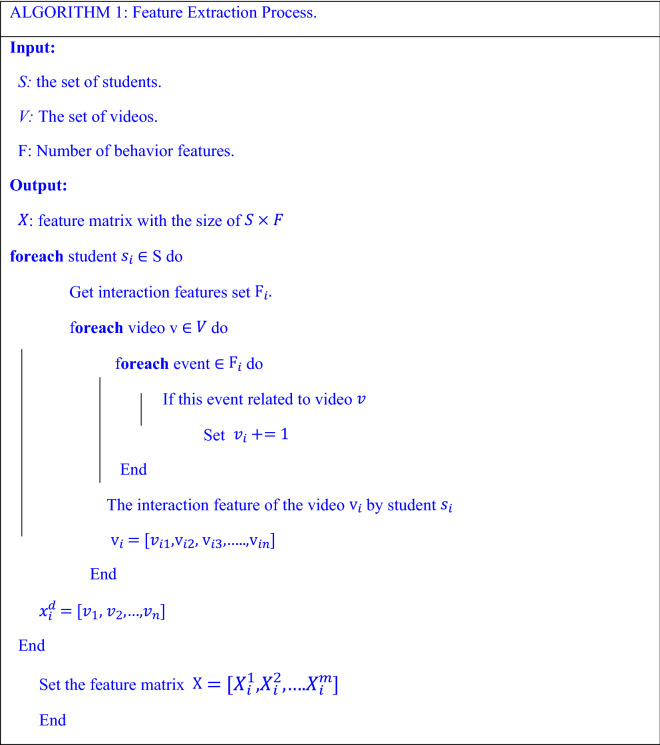

**Table 3:** The basic information of datasets

Name of course	Mining of massive datasets	Automata theory
Time frame	7 weeks	6 weeks
Modules lectures	15	6
Videos	94	22
Students	6711	6390
Clickstream	1,821,041	675,706
Quizzes	15	6

### Heterogeneous knowledge graph (HKG) representation

Based on the historical online course data, a heterogeneous knowledge graph structure was constructed to model the relation among different entities (students, videos, knowledge concept) as $G=\{V,S,{X}_{v},{X}_{s},E\}$ represents a knowledge graph $G$ comprising the set of $m$ video nodes $V$, set of $K$ student nodes $S$, video features ${X}_{v}\epsilon {\mathbb{R}}^{{d}_{v}}$, and student interaction features ${X}_{s}\epsilon {\mathbb{R}}^{{d}_{s}}$. Since the main goal is to students classify, *E* represents relationship between students as edge, which can be extracted as the adjacency matrix ${A}_{i,j}\in {\mathbb{R}}^{k\times k}$, where $k$ is the number of the student nodes. ${a}_{i,j}$ is equal to 1. If there are links between student nodes, otherwise, ${a}_{i,j}$ is equal to 0. Furthermore, to link students who have similar learning behavioral patterns and knowledge concepts, first, a G-graph was built with each student and video as nodes. Thus, we formed different types of links between student nodes based on the video which they watched as edges. Besides, video nodes were connected in the graph based on the knowledge concept between them. Thus, the main knowledge concept related to the videos was identified as a self-supervisory signal and employed it as a link (edge) between students. It can be noted that these concepts are relevant implicit knowledge in the videos which do not need prior-labels and costs. As long as the graph is constructed, the hidden features are iteratively transformed and propagate information across neighbors to capture k-hops away from structures in the graph for each student. Thus, we employ a relational graph convolution network as a representation learning model to learn the low-dimensional representations of the entities in a heterogeneous view, as shown in Fig. 1, which can use the representations of the relationship between students to classify them.

### Graph convolutional networks for HKG representation learning

According to the definition of heterogeneous information network (HIN) [32], heterogeneous knowledge graph (HGK) is denoted as $G = (N, E, \mathrm{\rm X})$ which consists of node set *N*, edge set $E$ between nodes, and $X$ is the feature matrix of all student nodes. To classify student nodes in graph $G$, we feed feature matrix of student nodes with $\mathrm{\rm X}\in {\mathbb{R}}^{k\times d}$ and adjacency matrix $A\in {\mathbb{R}}^{k\times k}$ that denotes the topological structure of graph $G$ of students to the graph convolutional networks (GCN) as the input. The propagation rule between layers is applied as

1 $$ H^{{\left( {l + 1} \right)}} = f\left( {H^{\left( l \right)} ,A} \right) = \sigma \left( {\tilde{D}^{{ - \frac{1}{2}}} \mathop A\limits^{ \vee } \tilde{D}^{{ - \frac{1}{2}}} H^{\left( l \right)} W^{\left( l \right)} } \right), $$

where $\tilde{A }=A+{I}_{n}$ indicates adjacency matrix corresponding to nodes relation with self-connections, ${I}_{n}$ is the identity matrix. $\tilde{D }=\sum_{j}{\tilde{A }}_{j}$ is the degree matrix of graph G, and ${W}^{\left(l\right)}$ is a layer-certain $l$ trainable weight matrix. ${H}^{\left(l\right)}$ represents the feature matrix given by $lth$ layer; and the ${H}^{\left(0\right)}={X}_{f}$ where ${X}_{f}$ represents feature matrix input into the first GCN layer. Here, $\sigma \left(.\right)$ is an element-wise non-linear activation function such as $ReLU\left(a\right)=\mathrm{max}(0,a)$ and ${H}^{\left(l+1\right)}={Z}_{y}^{n}$ the output of the last GCN layer that indicates the label prediction for all *n*th node, and $y$ is classes number.

In GNNs, the convolutional layer aggregates a node neighboring nodes information and creates a higher level of node embedding vector by Eq. 2:

2 $$ h_{i} = \sigma \left( {\mathop \sum \limits_{j} \frac{1}{{v_{ij} }}h_{j} W} \right), $$

where node $j$ is node *i*’s neighbor.

The proposed model is graph-based semi-supervised learning of node classification task by GCN [i.e., the graph structure (edges of the graph) enables the GCN model to use a set of static training nodes to predict unlabeled nodes]. On the basis of that, the problem of student’s performance classification can be formatted in graph-based semi-supervised learning as a node classification task, where label information is smoothed over the graph via some form of explicit graph-based regularization. Therefore, the proposed model adopts a multiple-layer graph convolutional network which was presented in [33] because of its capability to employ deep learning on structure data of graphs by relying on an effective variant of CNN which performs directly on graphs. More specifically, it should be noted that a GCN cannot propagate the label information adequately into the whole graph with only a few labels, because refined employment of Laplacian smoothing may mix vertical features of various categories and render them unknown while training [34]. Therefore, GCN requires a considerable quantity of labeled data. The most stable nodes for each class are selected and then added to the training set. Increasing the number of static training nodes improves accuracy of predictions.

Furthermore, the main idea of the proposed model is that we want to learn a better set of latent features to understand students' performance and to better classify them, rather than only using his features alone. The GCN model has two used layers. The first layer is employed to consolidate the features extracted of all the student (i.e., the 1-hop neighbors for a student ${S}_{i}$ that takes into account all videos watched). Then, using the second layer would furthermore incorporate the 2-hop neighbors which would involve information from all the neighbors of ${S}_{i}$ who have the same knowledge concepts, and consequently provide an additional context to learn a more comprehensive embedding for student.

Thus, to boost the performance of GCN more accurately and apply semi-supervised learning, the spectral convolution on the graph is employed [35] as

3 $${g}_{\theta }\times x=U{g}_{\theta }{U}^{T}x,$$

where ${g}_{\theta }=\mathrm{diag}(\theta )$ indicates a filter on spectral domain, $U$ is an eigenvector matrix of the normalized graph Laplacian $L=U\Lambda {U}^{T}$ with $\Lambda$ is eigenvalue matrix of $L$. Equation 3 is a complex computation in this type of spectral convolution. Therefore, to reduce that, the Chebyshev polynomials [36] are applied

4 $${g}_{\theta }\times x\approx U\sum_{k=0}^{k}{\grave{\theta }}_{k}{T}_{k}\left(\stackrel{\sim }{\Lambda }\right){U}^{T}x= \sum_{k=0}^{k}{\grave{\theta }}_{k}{T}_{k}\left(\stackrel{\sim }{\mathrm{L}}\right)x,$$

where ${T}_{k}\left(\bullet \right)$ indicates Chebyshev polynomial of *k*th order. With rescaled $\Lambda$ by $\stackrel{\sim }{\Lambda }=\frac{2\Lambda }{{\lambda }_{\mathrm{max}}}-{I}_{N}$, ${\lambda }_{\mathrm{max}}$ represents the largest eigenvalue of $L$. Thus, the $\stackrel{\sim }{L}=\frac{2L}{{\lambda }_{\mathrm{max}}}-{I}_{N}$. The expression in Eq. 4 depends only on nodes that are maximum at K steps away from the central node (Kth-order neighborhood, [36] employ this K-localized convolution to define a convolutional neural network on graphs). According to [33], the linear formulation of a GCN approximates ${\lambda }_{\mathrm{max}}=2$ and *k* = 2, and under these approximations, Eq. 4 is simplified into Eqs. 5, 6, and 7

5 $$ g_{\theta } \times x \approx \mathop {\grave{\theta}}_{0} { }x - \mathop {\grave{\theta}}_{1} D^{{ - \frac{1}{2}}} AD^{{ - \frac{1}{2}}} x, $$

with parameters $\mathop \theta \limits^{`}_{0} and \mathop {\grave{\theta}}_{1}$ can be further simplified by $\mathop \theta \limits^{`}_{0} = \mathop {\grave{\theta}}_{1} = \theta$

6 $$ g_{\theta } \times x \approx \theta \left( {{ }I_{N} + D^{{ - \frac{1}{2}}} AD^{{ - \frac{1}{2}}} } \right)x. $$

The ${I}_{N}+{D}^{-\frac{1}{2}}A{D}^{-\frac{1}{2}}$ normalized to [0,1]

7 $$ g_{\theta } \times x \approx \theta D^{{ - \frac{1}{2}}} AD^{{ - \frac{1}{2}}} x. $$

In this way, nodes can be selected more accurately and possible error classification is reduced. Thus, the forwarded proposed model takes the simple form of

8 $${Z}_{k}^{n}= f\left(X,A \right)={f}_{\mathrm{softmax}}\left(\tilde{A } {f}_{ReLU}\left(\tilde{A }X{W}^{0}\right){W}^{1}\right),$$

where ${W}^{0}$ is weight matrix of input-to-hidden for a hidden layer, ${W}^{1}$ is weight matrix of hidden-to-output. $X$ is feature matrix for all nodes, and $\tilde{A }$ is normalized matrix calculated by $\tilde{A} = \tilde{D}^{{ - \frac{1}{2}}} \mathop {{ }A{ }}\limits^{ \vee } \tilde{D}^{{ - \frac{1}{2}}}$, and $Z_{k}^{n}$ is the final output for output layer with the SoftMax function that indicates the label prediction for the *i*th node belonging to the class ${y}_{i}\in \left|Y\right|$.

To train the proposed model to classify, loss function as the cross-entropy was employed to evaluate error over limited labeled instances as

9 $${\mathcal{L}}_{\mathrm{semi}}=\sum_{i\in {D}_{l}}{\sum }_{f=1}^{F}{Y}_{lf}ln{Z}_{lf},$$

where ${D}_{l}$ is a set of labeled nodes, and $F$ is the output feature dimension, which indicates class counts, and ${Y}_{lf}$ is ground truth.

In the context of semi-supervised node classification tasks, a classifier targets to learn samples D from a set of N training. These samples are separated into an unlabeled set ${D}_{u}={\left\{{n}_{i}\}\right.}_{i}^{{N}_{u}}$ and a labeled set ${D}_{p}={\left\{{(n}_{i}{,y}_{i})\}\right.}_{i}^{{p}_{l}}$, where ${y}_{i}$ is one label for C classes. The prediction for unlabeled Nodes is unsupervised learning that we aim to perform semi-supervised for these ${N}_{u}$ unlabeled samples, assuming that a label $y$ is available for these samples. The pseudo-code of proposed model during training and optimizing is shown in algorithm 2.
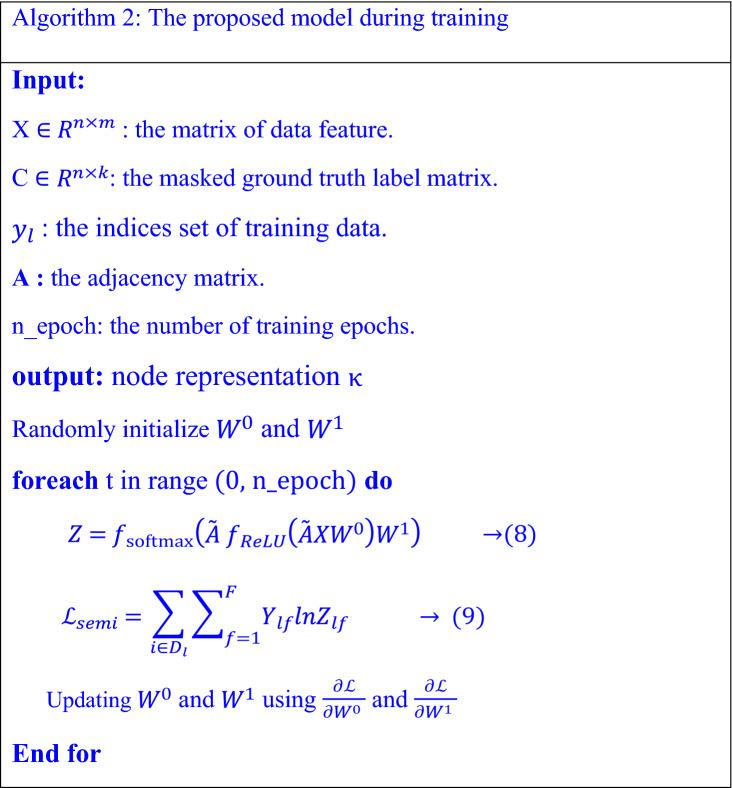


## Experiments and performance evaluation

In this section, the experiments are conducted on two different datasets to evaluate prediction accuracy and weighted-F1 score of students’ performances on each class (4 class levels) and semi-supervised node classification.

### Dataset description

The datasets employed in this study were collected by the Center for Advanced Research Through Online Learning (CAROL) [37] at the University of Stanford, which were offered over the SELF-PACED and Fall 2016. Each of these two courses falls under computer Science topics. The first course is “Mining Massive datasets”, and the second one is “Automata Theory”. Each course includes a mixture of topics which are divided into multi-modules of videos related to the specific concept of knowledge and homework on sequence weeks and a final exam. Table schema for each course includes three tables which are “events Extract”, “video Interaction”, and “activityGrade”. In the video interaction table, each record represents students' behavioral data with any events of video such as (play, pause, speed change, and so on.), student/video identification information, and the course. Homework assessment grades are recorded in “activity grade” table, and the other events related to the course are stored in “eventsExtract” table (e.g., click on website, problem, go to the discussion, and extra events). Table 2 exhibits a summary of the basic information about the data of the two courses after pre-processing. This is done by pre-processing the dataset and relevant features extraction for students (behavioral data selected), video events (e.g., play, pause, seek fore/backward, stop, load), and related knowledge concept. We noticed that most of the students did not interact with all the videos sequentially for all weeks. They sought to understand or absorb specific knowledge through their engagement of some concepts of related videos.

### Data pre-processing

Before the data become fit to be analyzed, additional data preparation techniques were conducted, such as removing the empty rows (rows with null values), labeling, and encoding. The error-free data were obtained. We also converted the string variable to be assigned as a numeric variable to fit analysis processes.

Since, the scenario followed is to classify students into four different levels (not only pass and drop out) according to their interaction with course videos of the previous weeks and their correlation with peers in the same concept of knowledge. Therefore, when the proposed model is trained on the current data, there is no consistency between ground truth and the class label that the model has predicted (four classes), because the real data typically contain the final score of the student (1 is pass, or 0 is failure). Besides, when evaluating the student's performance in a specific week, it also lacks the result of the student's performance. Accordingly, one of the requirements is data labeling to different classes that can be adopted as ground truth to be compared with the class label that the model predicted. Data labeling is an important manner of data pre-processing for machine learning techniques, in which both input and output data are labeled for classification to provide a learning foundation for future data processing.

As part of this work, we have used data programming [38], a model for programmatic creation and dataset training, which enables experts of the domain to more rapidly train machine learning systems. In data programming, instead of hand-labeling each instance, users provide a set of heuristic rules called labeling function that provides a label for each point group of the training data.

A labeling function draws a pattern which users enable to present to their model, which is simpler to encode than as a set of hand-labeled instances. Labeling functions do not require to have perfect accuracy or recall; thus, the effect of a feature at the end of the performance is based on the training set and on statistical attributes of the model [39]. The labeling functions require to set certain conditions and rules that can seamlessly synthesize labeling functions. To achieve this, we investigated some of the features that involve video-related features and the effort-related feature (performing week exams and a number of attempts for each exam) to conclude whether the student is engaged or not.

Many previous studies have proposed different engagement metrics. Koster et al. (2016) proposed employing interaction frequency with a tablet-installed app (opening, closing application, accessing the material, and browsing a questionnaire) to identify students’ engagement. Similarly, Ramesh et al. (2013) employed three key interaction-related features to investigate students' engagement level in MOOCs setting. They considered the number of posts in forums, a number of content views, and binary indicators of assignment completion as metrics to determine the engagement level of students in a three-level model. In a similar way, several metrics were presented by Kim et al. (2016), such as the number of visits and the total time spent in LMS as proxy features as indicators for academic performance in asynchronous online discussion. The metrics of engagement employed in the literature provide an inspiration to the type of metrics that can represent the engagement of students in an online learning environment. In line with that, we propose interaction-related features and effort-related features as proxy metrics for data labeling based on the engagement level of students.

Interaction-related features are defined as a sum of averages of video-viewing time relative to the actual video time, whereas effort-related features represent the effort committed by the student to perform the week exam and the count of attempts times for taking the exam. For interaction-related feature (watching videos rate), watching rate (${WR}_{V}$) was employed for each video watched by student *S*, which was calculated based on the watched event on its time duration as follows:

10 $$ WR_{V} = \frac{{W_{t} }}{{v_{t} }}, $$

where $W_{t}$ is the spent time of student $S$ to viewed video $V$ and $v_{t}$ is time duration of video. Second, $WR_{s}$ equation mathematically quantizes videos viewing. Furthermore, it can estimate an average of the viewed videos for a single student as follows:

11 $$ WR_{S} = \frac{{\mathop \sum \nolimits_{i = 1}^{m} WR_{v} }}{m}, $$

where $\sum_{i=1}^{m}{WR}_{v}$ is sum of the averages of the viewing of student *S* of all course videos, and $m$ is the number of the course videos. Based on the ${WR}_{S}$ values, it is possible to know the average of watching the videos of the entire course for each student.

Based on these two metrics and the formulation of the classification modes mentioned in the second section (Table 1), we construct the label function. The pseudo-code of label function is shown in algorithm 3. The label function was implemented on the dataset to automatically label data.
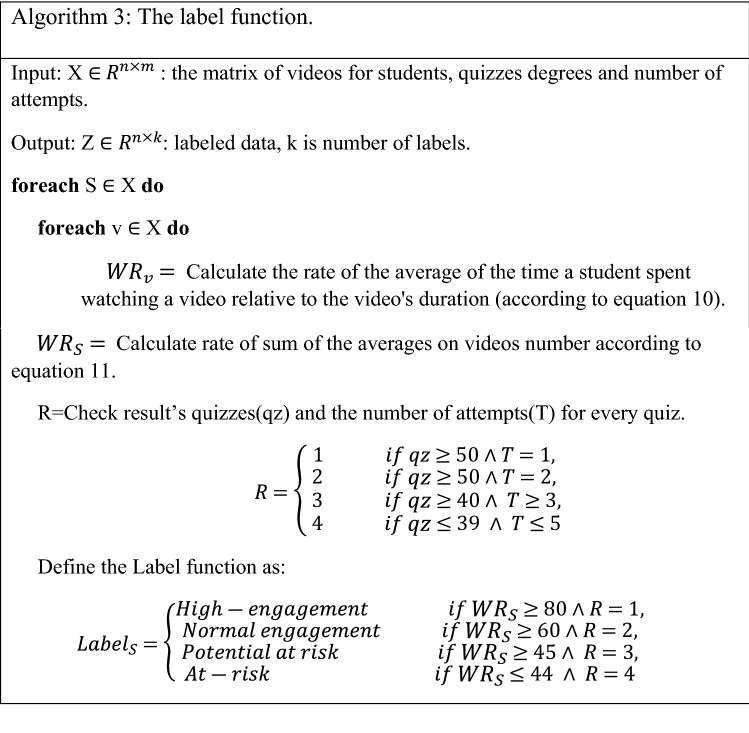


According to the outcomes of the metrics proposed. Therefore, each student was marked by a label indicating the level of his engagement. For example, students who had the highest participation level were labeled in the high-engage class. Likewise, the least participating students were labeled in the likely dropout class. The data labeled column will be used as ground truth to evaluate the class label that the model predicted (four classes) during application on the testing data and the new data.

## Experimental settings

**Fig. 2:**
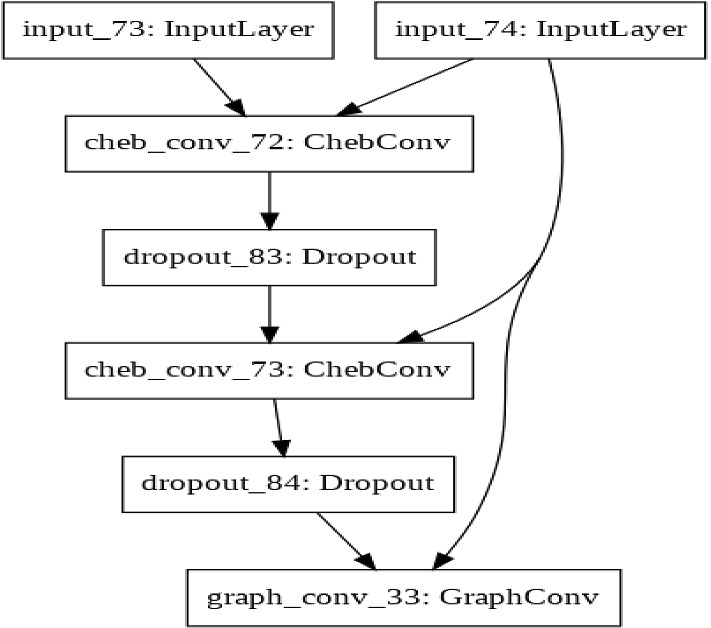
Diagram of GCN trainable model

By the heterogeneous knowledge graph constructed to model the relation of course videos, students, and knowledge concept, the symmetric adjacency matrix *A* was extracted using edges between the nodes and is fed into GNN models alongside node features matrix. The proposed model was evaluated for semi-supervised node classification tasks. Therefore, the labels are encoded and converted to a one-hot encoding. The spektral package [40] was used to implement the model and was applied with TensorFlow and Keras packages. The hyper-parameters of the model are tuned according to [33], where the same dataset splits are chosen with the validation set of 500 labeled examples for hyperparameter optimization (dropout rate for all layers, *L*2 regularization factor for the first layer, and the number of hidden units). The grid search method is used for hyperparameter tuning where we built a model for each possible combination of all of the hyperparameter values given, assessing each model, and deciding the structure which produces the best results. Many parameters were deployed to optimize performance of model output and employ the hyperparameter optimization approach to find the best model. The proposed model ran with 200 epochs with a learning rate set to 0.001 with Adam optimizer and the batch size adapted with the whole graph size. Otherwise, the graph would be shuffled. Weight regularization and dropout techniques were adopted to avoid over-fitting and to regularize the network. Besides, the TensorBoard in the callbacks called to monitor training and validation accuracy and loss. The hyper-parameters were optimized on the dataset “*Mining of Massive Datasets”* only and used the same set of parameters for the dataset “Automata Theory”. Figure 2 shows the structure of the GCN model which was used as trainable for both datasets. During training the model on both datasets, 20 labeled nodes per class were employed for training, and 500 labeled samples were randomly selected for the validation set, and 1000 labeled samples were selected as test nodes to evaluate the model, as shown in Table 4. The label rate refers to the ratio of labeled nodes to the total number of nodes. The transductive setting was implemented, which means that the whole graph was fed to the model during training and testing. Bool masks were employed to split training, validation, and testing of data, and were set to *sample weight* argument.

**Table 4:** Summary of datasets statistics used in experiments.

Name of course	Mining of massive datasets	Automata theory
Nodes	6805	6412
Edges	22,159	11,076
Classes	4	4
Features	202	152
Training nodes	80	80
Validation nodes	500	500
Testing nodes	1000	1000
Label rate	0.011	0.012

**Fig. 3:**
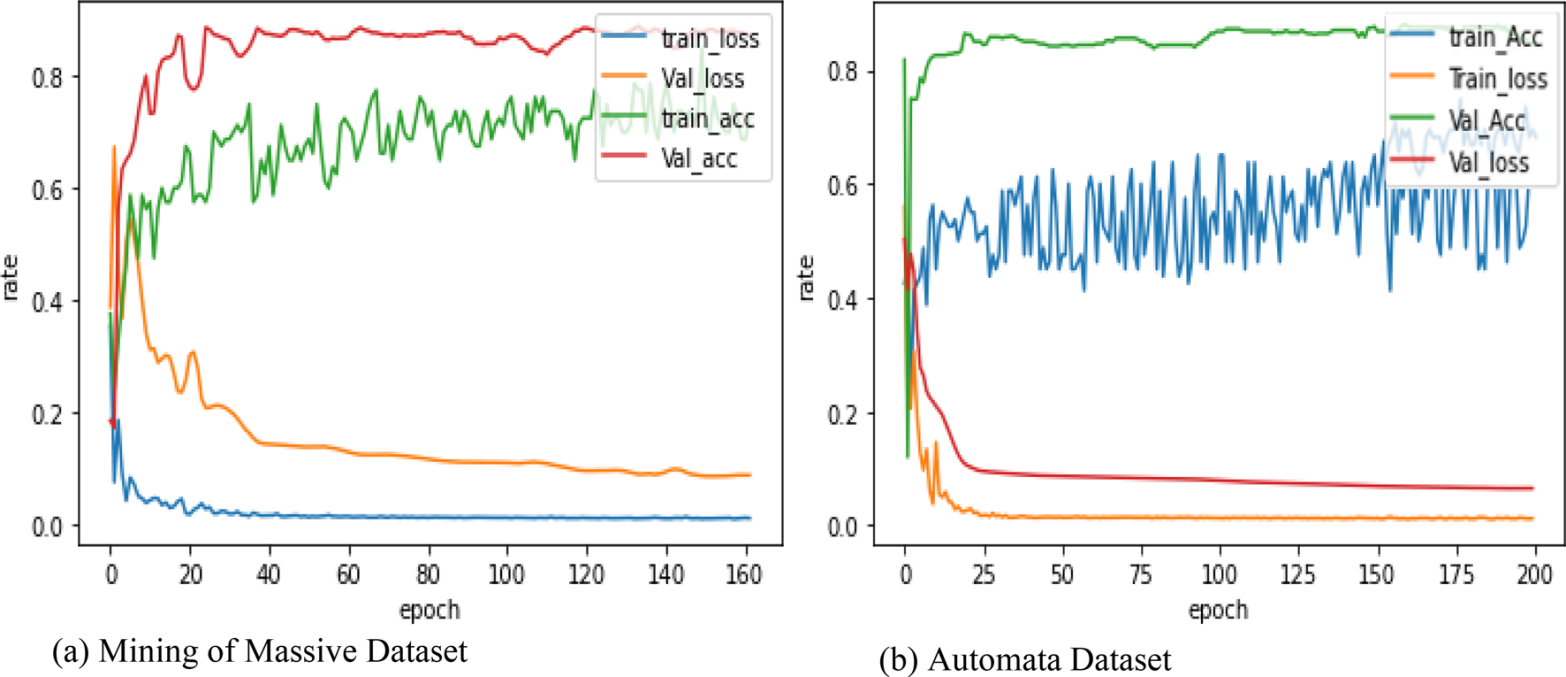
Accuracy and loss plots visualization during training the proposed model by validation and train dataset.

During training, the proposed model was performed on 200 epochs due to the Early Stopping function implemented with patience of 20. This means that training will stop once validation loss stops at a decrease of 20 sequential epochs. The history object was employed from the fit function, which can store history values of accuracy and loss to plots between training and validation, which can enable us visualize performance of model. At the end of training the model, we obtained the suitable model, because accuracy of training was 84%, and loss score was 26% in dataset 1, while the rate of accuracy was 82% and loss score was 29% in dataset 2, as shown in Fig. 3.

## Discussion results and evaluation of the model

To verify efficiency and accuracy of the proposed model on semi-supervised learning tasks for prediction of students' performance, the weighted-F1 score which is the harmonic mean of recall and precision was used. As mentioned before, the dataset involves four different labels to detect the level of students' performance through their engagement with the course, namely “High_engagement”, “Normal_engagement”, “At-risk”, and “Potential_At-risk”. According to the classification report, Table 5 shows classification metrics precision, recall, and f1-score, which were calculated by true and false positives, and true and false negatives on a per-class basis. The macro average of F1-score was 80%, and accuracy rate was 84% and 82% in both datasets, respectively. Therefore, the model can classify students into different levels based on their learning styles. The t-SNE algorithm [41] was employed to visualize the first hidden layer representations as 2D during training the model on both datasets.

**Table 5:** Classification results of the proposed model for both datasets

Course name/classes and avg	Mining of massive datasets				Automata theory			
	Precision	Recall	F1-score	Support	Precision	Recall	F1-score	Support
At-risk	0.96	0.98	0.97	705	0.97	0.93	0.95	594
Normal-engagement	0.76	0.72	0.74	62	0.74	0.7	0.71	79
High-engagement	0.83	0.78	0.81	149	0.78	0.91	0.84	192
Potential-At-Risk	0.75	0.66	0.7	84	0.73	0.72	0.72	135
Accuracy			**0.84**				**0.82**	
Macro avg	0.82	0.78	0.80	1000	0.80	0.81	0.80	1000
Weighted avg	0.91	0.90	0.90	1000	0.88	0.87	0.87	1000

**Fig. 4:**
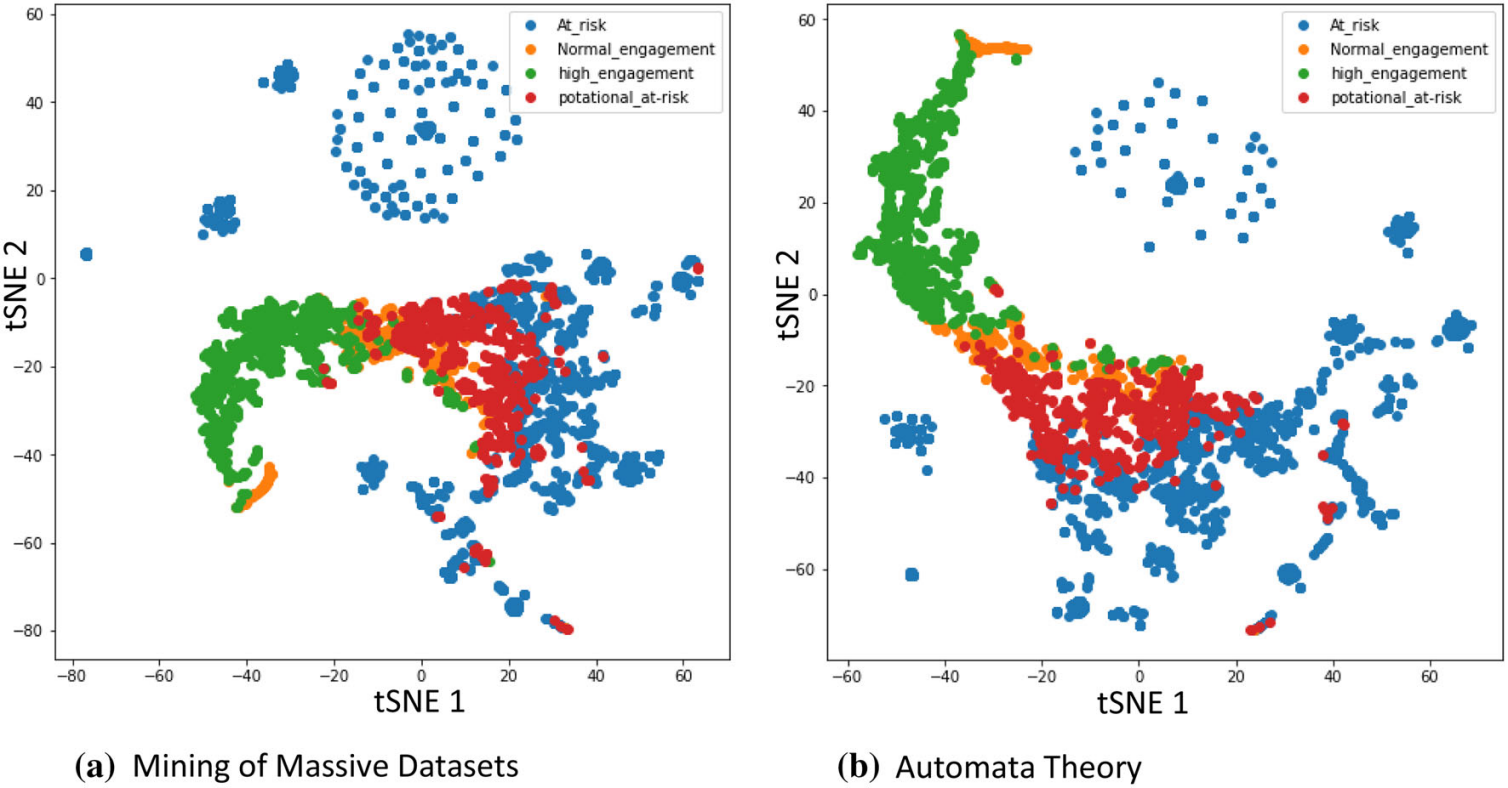
T-SNE visualization of hidden features for a proposed model on both datasets to classification the students’ performance with different clusters

Each node (or student) was represented as a point in the plot, while each type of class was marked by a different color. It can be noted form Fig. 4 that the data of each class were distributed and allocated more clearly, which illustrates the discriminative ability of the proposed model to conduct graph node representation and semi-supervised classification tasks.

To further evaluate efficiency and generalizability of the proposed model, performance of the model was compared to machine learning methods as the semi-supervised node classification task for student performance classification on the test data. In baseline methods, we concatenated the course video features and students’ features (i.e., behavioral data) and normalized them by min–max scaling to the 0–1 range and transferred them into a single vector, which was fed for training and prediction of baseline methods. These baselines are as follows:

- In DNN, two layers were used in the training each of which had 128 units and *ReLU* function, and some parameters, such as learning rate at 0.001 with Adam optimizer. Dropout layer and the output layer with SoftMax function were set.
- A support vector machine (SVM) [42] with radial basis function kernel (RBF) was set to regularize the parameter set to 1 with parameter cost and gamma. Therefore, a model constructed a hyperplane to distinguish samples.
- Random Forest [43] is an ensemble learning method of classification and regression which suits many decision trees on different sub-samples of training data and uses the mean to improve prediction accuracy and prevent over-fitting.

**Fig. 5:**
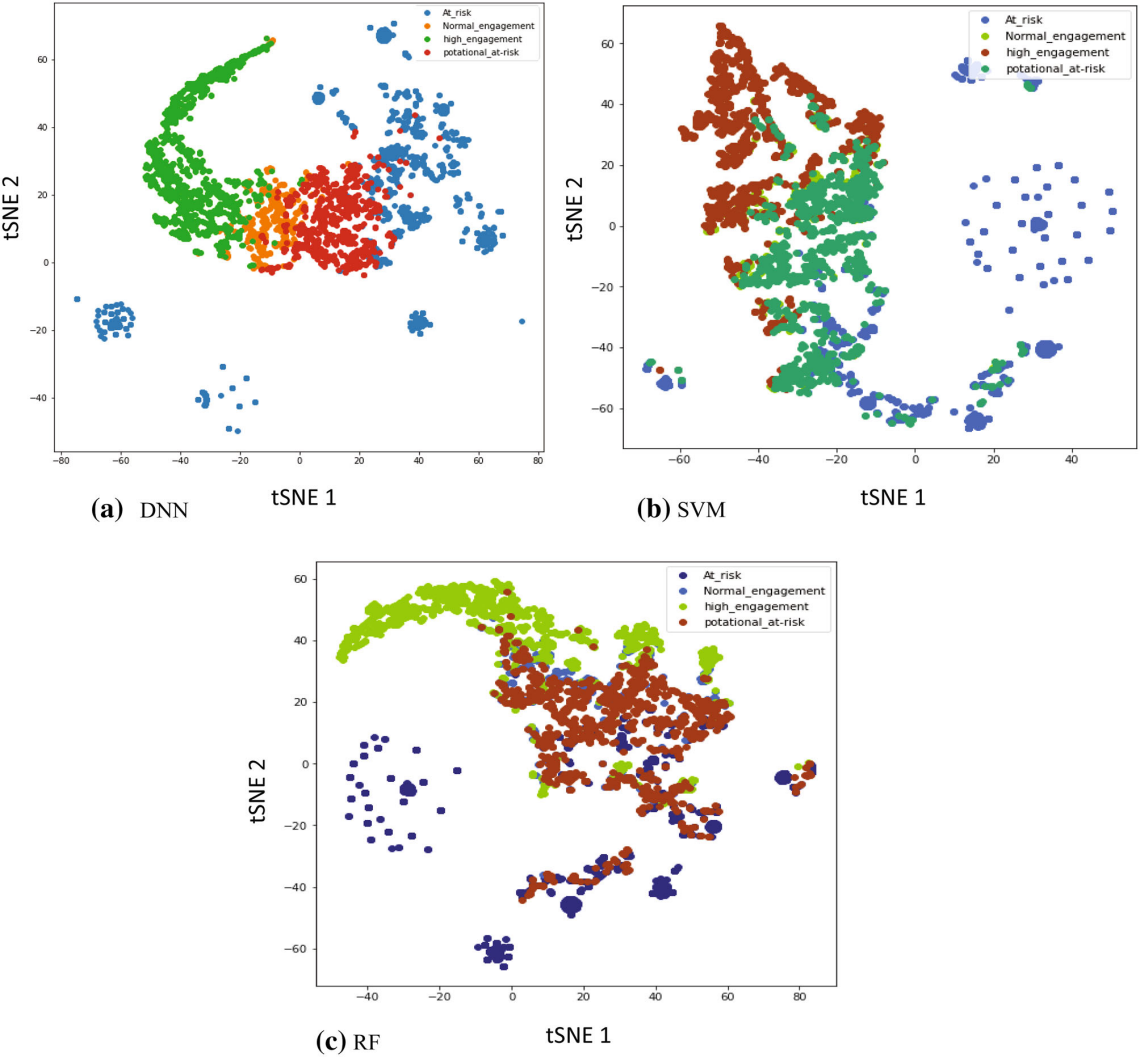
T-SNE representation of baseline models outputs to classification the students’ performance into different clusters on “mining of massive datasets” course.

These methods were employed in previous studies [44, 45]. They achieved excellent performance to predict students’ performance in various perspectives. In previous studies, SVM achieved an accuracy rate of 79.95–89.14% in [46], and Random Forest achieved 81.25–96.01% in [44, 45], while in [47], DNN outperformed SVM and Random Forest. Our finding is compared to the results of baseline methods in terms of accuracy and f1-score as exhibited in Table 6. Our proposed model has higher accuracy of prediction of students' performance, whereas baseline methods did not achieve good prediction in terms of accuracy and f1-score of 76.9–82%, whereas RF achieved an accuracy rate of 79.3–78.3% and an f1-score of 74–78%. DNN outperformance rate was 82–81.9% in accuracy and 77% in f1-score for both datasets’ score. SVM achieved an accuracy and fl-score of 78.6–74%, respectively. According to Table 6, the proposed model has an average accuracy of 3.5% higher than DNN, and 6% higher than Random Forest. The best results are marked as bold. In addition, the t-SNE algorithm was employed for baseline model outcomes to visualize the similar student nodes clustered together in the graph on “Mining of Massive Datasets” dataset, as shown in Fig. 5. The t-SNE algorithm explores patterns in the data by identifying observed clusters to group local data points closer to each other based on the similarity of data points with multiple features. Figure 5 generated by t-SNE contains some points that are clustered with the incorrect class; however, most of these points correspond to distorted digits, many of which are difficult to identify.

**Table 6:** Comparison results of proposed model and baseline models

Course name		Mining of massive datasets				Automata theory			
Methods	Classes and avg	Precision	Recall	F1-score	Support	Precision	Recall	F1-score	Support
The proposed model	At-risk	0.96	0.98	0.97	705	0.97	0.93	0.95	594
	Normal-engagement	0.76	0.72	0.74	62	0.74	0.7	0.71	79
	High-engagement	0.83	0.78	0.81	149	0.78	0.91	0.84	192
	Potential-At-Risk	0.75	0.66	0.7	84	0.73	0.72	0.72	135
	Accuracy			**0.84**				**0.82**	
	Macro avg	0.82	0.78	0.80	1000	0.80	0.81	0.80	1000
	Weighted avg	0.91	0.90	0.90	1000	0.88	0.87	0.87	1000
DNN	At-risk	0.9	0.92	0.91	705	0.9	0.94	0.92	594
	Normal-engagement	0.74	0.72	0.73	62	0.78	0.76	0.77	79
	High-engagement	0.89	0.73	0.8	149	0.75	0.78	0.75	192
	Potential-At-Risk	0.61	0.73	0.66	84	0.62	0.74	0.67	135
	accuracy			**0.82**	1000			**0.81**	1000
	Macro avg	0.78	0.77	0.77	1000	0.76	0.80	0.77	1000
	Weighted avg	0.86	0.86	0.86	1000	0.82	0.86	0.84	1000
SVM	At-risk	0.89	0.85	0.86	705	0.87	0.85	0.86	594
	Normal-engagement	0.67	0.64	0.66	62	0.51	0.67	0.58	79
	High-engagement	0.76	0.74	0.75	149	0.76	0.79	0.77	192
	Potential-At-Risk	0.69	0.7	0.7	84	0.67	0.69	0.68	135
	accuracy			**0.79**	1000			**0.77**	1000
	Macro avg	0.75	0.73	0.74	1000	0.70	0.75	0.72	1000
	Weighted avg	0.84	0.80	0.82	1000	0.79	0.80	0.79	1000
Random Forest	At-risk	0.88	0.87	0.88	705	0.87	0.93	0.9	594
	Normal-engagement	0.67	0.74	0.71	62	0.51	0.67	0.58	79
	High-engagement	0.86	0.8	0.83	149	0.71	0.79	0.75	192
	Potential-At-Risk	0.67	0.73	0.7	84	0.7	0.79	0.74	135
	accuracy			**0.79**	1000			**0.78**	1000
	Macro avg	0.77	0.78	0.78	1000	0.69	0.79	0.74	1000
	Weighted avg	0.84	0.83	0.85	1000	0.78	0.86	0.82	1000

Figure 4 constructed based on the proposed model is significantly better, since it models many of the nodes of each class fairly close together, but none of the classes are clearly separated. In contrast, the t-SNE in Fig. 5 shows much distribution of data points. Moreover, data in Fig. 5 are not distributed or allocated more clearly for each class when compared to Fig. 4, which explains the discriminative capability of the proposed model to conduct graph node representation and semi-supervised classification tasks.

For further investigation about the effectiveness of the proposed model, below is an extensive comparison between our results and results of the previous studies which used GCN to analyze MOOCS data in terms of accuracy and F1-score. In [48], attention-based graph convolutional networks model is used to predict students’ performance and detect at-risk students by analyzing students’ data from previous courses. That model achieved a percentage of F1 scoring 70–78% for detecting at-risk students based on data of the whole courses and prior courses. In the same way, the paper [49] employed GCNs as an approach to predict students’ performance in the next semester based on their previous final exam results. The model was able to predict an average accuracy of 81.5%. Similarly, [50] proposed a model based on GNNs to predict students’ score level on each question in interactive online question pools. They modeled students' performance in interactive online question pools as a node classification problem on a heterogeneous network (questions, students). The model achieved a relative prediction accuracy of 66%.

In the same context, [51] is the most similar work to our work. Authors studied the impact of video-viewing behavior on learners’ performance to predict whether or not a learner will succeed. They did not focus on clickstreams learners made, but they studied the pedagogical sequences in which those clicks were made. Thus, the text GCNs’ model to predict learners’ performance is used. Text GCN results achieved an average accuracy of 67.23%, while our model achieved better results in accuracy matrices, which ranged from 82 to 84%.

Furthermore, the most important advantage of our model is its ability to predict student’s performance based on the weekly or the entire course data, in addition to classifying students according to their level of engagement, which enables those in charge of the course to perform a timely intervention.

In general, detecting at-risk students of dropout and predicting their performance are the primary tasks for early prediction and recommendation systems. The ability of a model to provide feedback for its predictions can increase its reliability.

Besides, the graph-based prediction model can visualize students' behavior changes over the course and give the interpretability of the prediction. Moreover, in learning-based models, the model learns the graph structure that helps to predict students’ performance. Thus, graph representations for the model output show high prediction performance that can provide insight regarding students’ status. For decision-makers, understanding the reasons behind predictions can help to reveal students’ knowledge status efficiently and logically.

In this respect, our proposed model was performed as learning-based through semi-supervised tasks. Students’ performance was analyzed in two steps. In the first step, based on students' association with the course videos, a knowledge graph was built between the students and the videos that were viewed by them as graph-structure data along with topology of graph which links the students who share the same concept of knowledge besides extracting important features of students’ interaction with concepts of knowledge of the course-related content. Accordingly, the model can update students’ status. In the second step, the performance of students was assessed. In this study, students’ engagement level was classified into four levels. The GCN model was used, which showed its efficiency better than baseline methods which do not take into account the structure of the knowledge graph. Wherever there is an update of the knowledge graph structure, students’ performance is evaluated. Therefore, using a heterogeneous graph representation, this proposed model will improve students’ acquisition of learning if compared to any statistical method relying solely on a static graph snapshot. More specifically, the proposed model can track students’ knowledge status better than the previous models, which do not consider knowledge graph structure.

The proposed model was applied on two different datasets. The second dataset was used as testing data. This indicates the generalizability of the model to other online courses data.

Overall, such a model could be employed during learning the online course to capture students' interaction data aggregated from previous weeks to classify students' engagement level and predict their performance in the next week. Instructors can know students who are likely to perform poorly and thus provide some intervention to them with the limited inherent resources in online systems.

## Conclusion and future works

Predicting students' performance and retention is vital in learning analysis field in MOOCs courses. In this paper, we proposed a model to predict students' performance based on their engagement with course videos and their performance on the assessments/quizzes conducted after. The Graph Convolutional Network (GCN) was adopted in model structure for semi-supervised learning tasks to formalize students' behavioral data in a more natural and intuitive way as a node classification problem. Students, course videos, and the interaction between them are represented as a heterogeneous knowledge graph. The proposed model utilizes the input of each layer data of vector representations and the adjacency matrix of the corresponding graph structure. Many extensive experiments were performed to assess the proposed model on different datasets. Results of the experiments showed that the proposed model outperformed baselines approach in terms of accuracy and f1-score. In addition, it is more efficient and feasible in classifying representation of students and in identifying students who are at-risk.

Overall, the proposed model can be applied to track students' performance. This may provide decision-makers and instructors with feedback about students who are at-risk of failing a course, which can help stakeholders to decide the right response/s that may augment the final outcomes of the course.

In future work, we will extend a heterogeneous knowledge graph by including other students’ interactions or by linking students’ behavior data from a social network for better predictions in online education, taking into account other factors such as assessment types. In addition, we seek to identify similarities between knowledge concepts of different courses and predict related courses for the future using hybrid methods based on Machine Learning Methods. We assume that this will be highly important in improving online learning systems.
